# African Forest Honey: an Overlooked NTFP with Potential to Support Livelihoods and Forests

**DOI:** 10.1007/s00267-018-1015-8

**Published:** 2018-03-08

**Authors:** Janet Lowore, Julia Meaton, Adrian Wood

**Affiliations:** 1 Bees for Development, 1 Agincourt Street, Monmouth, NP25 3DZ UK; 2 Huddersfield Business School, University of Huddersfield, Huddersfield, HD1 3DH UK

**Keywords:** Ethiopia, NTFP, Honey, Beekeeping, Livelihoods, Forest conservation

## Abstract

In parts of the developing world, deforestation rates are high and poverty is chronic and pervasive. Addressing these issues through the commercialization of non-timber forest products (NTFPs) has been widely researched, tested, and discussed. While the evidence is inconclusive, there is growing understanding of what works and why, and this paper examines the acknowledged success and failure factors. African forest honey has been relatively overlooked as an NTFP, an oversight this paper addresses. Drawing on evidence from a long-established forest conservation, livelihoods, and trade development initiative in SW Ethiopia, forest honey is benchmarked against accepted success and failure factors and is found to be a near-perfect NTFP. The criteria are primarily focused on livelihood impacts and consequently this paper makes recommendations for additional criteria directly related to forest maintenance.

## Introduction

Tropical forests are under threat from deforestation and degradation, caused by over-exploitation, logging, and conversion to other land uses (Megevand 2013; Bennett 2015; FAO 2016). Many different solutions have been explored, ranging from forest certification, statutory protection and community forest management, (Nelson et al. 2009; Kalonga et al. 2016). One strategy that gained traction in the 1990s focussed on the development of NTFPs as a means of making the forest pay its way and become a competitive land use for forest-fringe households (Peters et al. 1989). The idea is that if forests have value for local communities, they will be more inclined to maintain them. NTFP harvesting is described as “the practice of extracting economically valuable, non-timber forest products leaving the forests structurally and functionally intact”, (Nepstad and Schwartzman 1992). Evans (1993) called this the “conservation by commercialization” hypothesis.

Enthusiasm for this ‘‘win-win’’ solution to both poverty and deforestation resulted in significant research and action in the 1990s, with the initiation of development projects aimed at commercializing NTFPs to increase their value. These explored the potential of NTFPs as diverse as ant larvae in Indonesia and baobab juice in Malawi (Césard 2004; Kambewa and Utila 2008). However, many of these failed to achieve commercial viability and studies began to review the concept’s efficacy in safeguarding forests (Arnold and Pérez 2001; Kusters et al. 2006; Kusters 2009). A rich body of literature has identified and discussed various criteria for success and failure (Belcher and Schrekenberg 2007, Shackleton et al. 2011). The purpose of this paper is to present the case of one NTFP, African forest honey, and to consider it against these increasingly well-understood factors.

## African Forest Beekeeping

### Forest Beekeeping

This paper focuses specifically on forest beekeeping in Africa, which has features that distinguish it from other systems, such as large-scale bee farming or back-yard beekeeping (Wainwright 1989; Clauss 1992; Crane 1999; Bradbear 2008; Lowore and Bradbear 2015). African forest beekeeping utilizes the wild honey-bee population as a resource and does not involve manipulating this natural population. The bee colonies of the indigenous African honey bee *Apis mellifera* live within the forest and forage on nectar and pollen from a very wide range of floral species. Forest beekeeping involves the construction and siting of man-made beehives thus increasing the number of bee nest sites in a given area. Hives are made from locally available materials, sourced from the forest and vary in materials and design (e.g., where the entrance is), but the basic structure is a hollow cylinder. These are placed in forest trees and occupied by wild swarms of bees that are genetically undistinguishable from the wild population. Once or twice a year, depending on local seasonal cycles, beekeepers harvest honey comb, comprising two products in one, honey and beeswax.

Forest beekeeping is not honey hunting, which involves taking honey comb from wild-bee nests located in natural cavities (e.g., hollow trees and cavities in rocks). It also does not include the use of frame or top-bar hives, even if these are located in forests, since they are movable comb beekeeping systems that allow colony manipulation. In movable comb systems, beekeepers tend to focus on individual bee colonies as productive units with hives kept close to home, to manage and protect the colonies and hives. In forest beekeeping, the productive unit is the forest and its whole bee population and the system utilizes large forest areas that are unpredictable, undefensible and distant, hence making individual colony management impractial. Forest beekeeping is an extensive, low-input system (Bees for Development 2012, 2013a, 2013b).

Communities that engage in forest beekeeping in Africa depend heavily on income derived from selling honey and beeswax. In Mwinilunga, North West Zambia, 40,000 people depend on forest beekeeping using 60,000 ha of forest with 1000 tonnes of honey purchased from beekeepers in 2016 (Dan Ball Oct 2014, Oct 2016, personal communication). For many households in south-west (SW) Ethiopia, honey is the primary source of cash (Endalamaw 2005) and the number of hives is a wealth indicator, with anyone having 100 + hives considered rich. Unlike other wealth indicators, such as livestock, which the poor can rarely afford, many poor people do have small numbers of hives (van Biejnen et al. 2004). In Cameroon, honey accounts for just over half of household income for thousands of beekeepers (Ingram and Njikeu 2011). Beekeeping in Tanzania is so important (average annual export earnings of US$2.5 million) that it has a dedicated government department and 39,000 ha of forests set aside as bee reserves (Mwakalukwa 2016).

### Forest Beekeeping as an NTFP and its Relationship to Forest Management

Despite the interest in NTFP commercialization, forest honey seems relatively absent from the literature. For example, various notable NTFP research collections barely mention honey (Table 1).

**Table 1:** Notable NTFP research collections

Research	Reference to honey?
CIFOR’s comparative case studies of commercial production and trade of NTFPs (Ruiz Pérez et al. 2004)	None of the 61 cases concerned honey. Honey is mentioned once in a list of types of NTFPs
Riches of the forest series^a^ Fruits, remedies and handicrafts in Latin America (López et al. 2004) Food, spices, crafts, and resins of Asia (López and Shanley 2004a) For health, life, and spirit in Africa (López and Shanley 2004b)	Of 61 cases, one discusses honey harvesting in the Philippines
Forest products livelihoods and conservation series^b^	Of 61 cases, no case concerns honey
Volume 1 - Asia (Kusters and Belcher 2004) Volume 2 - Africa (Sunderland and Ndoye 2004) Volume 3 - Latin America (Alexiades and Shanley 2004)	
The literature resource of the Poverty and Conservation Learning Group (2016) http://povertyandconservation.info/en/bibliographies [accessed May 2016]	Out of 1800 articles only three mention honey in their titles or abstracts
Study of ten NTFP products from 18 marginalized communities in Bolivia and Mexico (Marshall et al. 2006)	Honey not mentioned
NTFPs in the global context (Shackleton et al. 2011)	In this 286-page book honey is mentioned six times (Zambian and Tanzanian honey exports)

^a^Part of CIFOR’s NTFP Case Comparison study—not all the same 61 cases

^b^Part of CIFOR’s NTFP Case Comparison study—not all the same 61 cases

However, honey has not been overlooked regarding livelihoods (Bradbear 2004). Concerning forests, beekeeping is often promoted as being forest-compatible but less frequently as a driver of conservation. Projects in Kilum-Ijim in Cameroon, Inyonga Forest in Tanzania, Mount Elgon in Uganda, and Selous in Tanzania have all included beekeeping ((Abbott et al. 1999, Turning our eyes from the forest. The role of Livelihoods Programme in changing attitudes and behaviour towards forest use and conservation at Kilum-Ijim Mountain Forest, Cameroon. Unpublished report for Birdlife International); Hausser and Savary 2002; IUCN 2012; Timmer and Juma 2005). In West Africa IUCN have supported beekeeping projects as components of their biodiversity conservation program (Arsene Sanon 2015, personal communication) and the Tanzanian government have a policy that promotes beekeeping to support forest conservation (Hausser and Mpuya 2004). However, the scientific rationale for these projects and evidence on their efficacy for forest conservation is limited (Ingram 2014) and the role of forest beekeepers as forest conservers is not understood. Mickels-Kokwe (2006, p 19) argues that in Zambia, *‘*‘‘the linkage between beekeeping and forest management has been considered to be strong. … the precise nature of this relationship, however, appears not to have been researched explicitly.” Bradbear (2009, p 58) concurs, ‘‘there has been little research to investigate how beekeepers make deliberate and conscious efforts to protect and conserve forests… this is an area of investigation that has been neglected.”

Literature on these relationships is limited, with few exceptions. De Jong’s study on forest conservation in West Kalimantan reported that honey had been traded since the 1930s and found that strong customary rules to protect honey forests existed among forest beekeeping communities, including one ‘‘Maté maté rule that no person except the owner of the honey tree may slash the forest within a radius of about 100 m… This rule ensures that the forest surrounding a honey tree is maintained and the habitat for bees is preserved (De Jong 2000, p 636).”

Most NTFP literature focuses on the income benefits of honey. For example, Ahenkan and Boon (2011) highlight the importance of NTFPs (including honey) for women’s empowerment in Ghana, but make no link to forest conservation. Some authors consider the promotion of beekeeping as a livelihood alternative to others that cause forest loss, but focus on farm-based, not forest beekeeping (Appiah et al. 2009; Tomaselli et al. 2012). Andrews (2006) and Labouisse et al. (2008) consider beekeeping as compatible with forest conservation but do not regard it as a driver. Within the wider beekeeping literature there is more insight into the conservation impacts of beekeeping. Clauss (1992) noted that Zambian beekeepers were worried about the impact of late fires^1^between August and October when trees and flowers of key nectar species are particularly vulnerable to scorching. Consequently, beekeepers advocate early burning to prevent such damage. Nshama (2003) reported that Tanzanian beekeepers sustained specific bee fodder plants, and Lalika and Machangu (2008) found beekeepers protected the forest around their hives and actively discouraged people from cutting timber. Endalamaw (2005) reported that 97% of beekeepers in SW Ethiopia were involved in at least one form of forest enhancement activity, including tree planting, preserving big trees, and protecting young ones; 34% helped to conserve the forest by lobbying or by entering into local agreements to reduce bushfires. Wiersum and Endalamaw (2013) also found that local forest governance arrangements in SW Ethiopia helped beekeepers support forest conservation that maximized honey production.

Bradbear (2009, p 58) draws evidence of the positive link between beekeeping and forest management from Congo, Benin, Zambia, and Tanzania and explains that ‘‘Apiculture’s unique feature as an activity is the fact that its continuation, through pollination, fosters the maintenance of an entire ecosystem, and not just a single crop or species.’’ In Cameroon, Ingram and Njikeu (2011, p 36) noted that ‘‘Beekeeping can contribute to environmental integrity because some beekeepers protect the forest’’, and Ingram (2014) later concluded that beekeepers rarely self-identified as active conservationists, but were so as a result of their pragmatic interventions. Finally, Neumann and Hirsch (2000, p 88) noted ‘‘that customary management for commercial NTFP production appears to occur least often in natural forests’’*’*but that ‘‘one example of commercial NTFPs that are managed in natural forests is honey and beeswax from beekeeping in Miombo woodlands in Africa.’’

## Methodological Approach

The paper considers how African forest honey can deliver positive outcomes for livelihoods and forests, using evidence from a project in south-west Ethiopia. The analytical process sought to:

- Identify and discuss known success and failure factors from the NTFP literature;

- Present a case where African forest honey is successfully commercialized;
- Compare forest honey against the known success and failure factors, with evidence from the case;
- Analyze the NTFP-PFM project’s^2^ support of forest honey regarding livelihoods and forest conservation and a reflection and critique of NTFP failure and success factors.

In order to assess the potential benefits of forest honey as a near-perfect NTFP, a case study analysis was undertaken (Yin 2011). The case study uses the honey- related evidence from the European Union (EU)-funded Non-Timber Forest Products-Participatory Forest Management Project in south-west Ethiopia. This case was selected because the length of the project intervention (2004 to present^3^) provides a wealth of documented information about changes and impact, and the authors have all been involved in the management of the project. The case study approach utilizes evidence from the five project area woredas, Anderacha, Bench, Gesha, Masha, and Sheko. Evidence was drawn from five principal project reports: van Biejnen et al. (2004), Abebe (2013), Bekele and Tesfaye (2013), NTFP-PFM (2013), and Lowore (2014). Two non-project reports, but covering the same area also provided important data (Endalamaw 2005) and (Bees for Development 2017).

## NTFP Commercialization, Success, and Failure Factors

The enthusiasm for NTFP commercialization led to a rich body of work, which latterly has been much more nuanced and grounded (Ros-Tonen and Wiersum 2005; Kusters et al. 2006; Belcher and Schrekenberg 2007; Sills et al. 2011). These, and other researchers, considered the features of NTFP trade that aid and hinder positive outcomes. It is important to clarify that these success and failure factors (Table 2) are considered in the relatively narrow sense of achieving commercialization, livelihood, and conservation outcomes and do not reflect broader benefits.

**Table 2:** Success and failure factors that have been shown to impact on the outcomes of NTFP trade

	References	T/L/C
‘‘Failure’’ factors		
Inferior: this concerns perishability, seasonality, and economic inferiority, i.e., product is rejected when incomes rise	Neumann and Hirsch 2000; Arnold and Pérez 2001; Sills et al. 2011	T
Substitutable: NTFPs can be easily replaced by manufactured or farmed alternatives, undermining sustainable trade	Arnold and Pérez 2001; Sills et al. 2011	T
Unmanageable: hard to manipulate quantity or quality of product	Belcher and Schrekenberg 2007; Sills et al. 2011	T
Elite capture: as a product increases in value, more powerful actors displace the original NTFP harvesters, and capture the benefits	Dove 1994; Sills et al. 2011	L
Poverty trap: decreasing prices force NTFP harvesters to collect more to earn the same	Belcher et al. 2005; Belcher and Schrekenberg 2007; Sills et al. 2011	L
Boom and bust: product is commercialized bringing income benefits until the resource becomes scarce, expensive, and ultimately replaced	Homma 1992	T
Over-exploitation: resource is over-harvested, causing depletion or extinction	Cunningham and Mbenkum 1993; Neumann and Hirsch 2000;	C
Diversity in the forest works against commercialization because not enough of the desired product	Neumann and Hirsch 2000	T
Product development, for new special products, can take a long time	Belcher and Schrekenberg 2007	T
‘‘Success’’ factors		
The natural resource base must be abundant to sustain viable trade	Cunningham 2011	T
Sustaining a market requires quality, quantity, and timeliness	Cunningham 2011	T
Adding value, if possible, can help grow and sustain beneficial trade	Cunningham 2011	L
Clear rights to land/not an open access situation aids positive outcomes	Neumann and Hirsch 2000; Cunningham 2011	L and C
Local self-sufficiency should not be undermined	Arnold and Pérez 2001; Cunningham 2011	L
Conflict resolution mechanisms are necessary	Cunningham 2011	T, L, C
Price incentives must be right	Cunningham 2011	T
Visionary champions make a difference	Cunningham 2011	T
Niche markets can reduce competition	Cunningham 2011	T
Strategic partnerships are important	Cunningham 2011	T
Additional factors		
Where earlier forms of trade precede an increase in demand, existing control systems may protect the resource from being plundered	Neumann and Hirsch 2000	T, L, C
The NTFP harvest must make the forest worth more than the alternative land use	Evans 1993	T, L, C
NTFP specialization can lead to forest modification, which may be inconsistent with the objective of maintaining biodiversity, but may be good for livelihoods	Neumann and Hirsch 2000; Ros-Tonen and Wiersum 2005; Belcher et al. 2005; Ruiz Pérez et al. 2004; Kusters et al. 2006; Belcher and Schrekenberg 2007; Sills et al. 2011	T, C
Biological characteristics of the NTFP determines likelihood and ease of sustainable harvest	Neumann and Hirsch 2000	C
Conservation logic, direct and tangible link between conservation action and benefit	Elliot and Sumba 2012	C

*T* aids or constrains commercialization and trade, *L* aids or constrains livelihood benefits, *C* aids or constrains conservation outcomes

Sills et al. (2011) analyze the evolution of the ‘‘conservation by commercialization’’ concept, and explain the swing from optimism to pessimism back to an ‘‘emerging middle ground.’’ They argue that the strengths of NTFPs are not so much in their promise as a ‘‘silver bullet’’ but in their diversity and collective contribution to rural livelihoods, and that forest modification is an important step in increasing incomes from NTFPs (Sills et al. 2011). They suggest that a holistic understanding is required and concur with Ros-Tonen and Wiersum (2005) that NTFPs are best understood in relation to the overall context of land uses and livelihood conditions. This paper makes no attempt to dispute these evident truths, yet chooses forest honey from among the diversity of NTFPs, as a focus for analysis. This approach is taken because the merits and features of forest honey as a commercially traded NTFP has been relatively unexplored, yet appears to offer considerable potential to deliver on both forest management and livelihood outcomes.

## Forest Honey Trade in SW Ethiopia

The NTFP-PFM project^4^ran from 2003–2013, and is now continuing with new funding and a new name.^5^The project was located in the moist montane forests in the Bench-Maji, Sheka and Kefa Zones of the Southern Nations, Nationalities, and People’s Regional State in south-west Ethiopia. These forests have high species endemism (Tadesse and Arassa 2004) and perform essential hydrological functions. They are highly valued by local people for domestic and economic purposes, and are the natural habitat of wild Arabica coffee (Hein and Gatzweiler 2006).

The forests cover approximately 3 m ha and form one of two major remaining forest blocks in the country (Sutcliffe et al. 2012), yet are experiencing a high rate of agricultural expansion and are exposed to considerable livestock and population pressure (Place et al. 2006). In some areas, the forest is highly modified to favor coffee management.

All natural forest has been state owned since the late nineteenth century creating a degree of alienation between local communities and the forest. A management vacuum resulted since the state is largely absent as a forest manager, although it does allocate forest land to private investors for plantation agriculture, exacerbating community alienation (Dessalegn 2011). There are some traditional tenure arrangements in the forest, called ‘‘*kobo*’’, discussed later, but these are unrecognized by government.

The potential of these forests to yield NTFPs such as wild coffee, spices, and honey was a major factor for the initiation of the project, which aimed to ‘‘maintain a forested landscape to support improved livelihoods of local forest-dependent communities and ensure the delivery of environmental services in a wider context.’’ The project sought to facilitate formal Participatory Forest Management (PFM) arrangements between local communities and government and to increase NTFP income-generating activities.

Honey is one of the most important forest products in the project area (Hartmann 2004; van Biejnen et al. 2004; NTFP-PFM 2013) and honey trade pre-dates the project. Westphal (1975) described the local economy as an ensete based mixed-cropping system including ensete, teff, barley, beans, and vegetables, but the most prominent cash income source was honey. Hartmann also explains, “Almost every payment is done during the honey harvest from the returns of honey marketing………… honey even can be used as payment instead of money” (Hartmann 2004, p 7). Honey is bought by local traders who supply the local and Ethiopia-wide *tej*^6^industry. A *tej* by-product is beeswax, much of which is exported, with Ethiopia annually exporting over 400 tonnes, the fourth largest global exporter (FAOSTAT 2005). A beeswax trader from the project area claimed he had exported 80 tonnes of beeswax each year for the last 20 years (Lowore 2014).

The project sought to achieve its objective by introducing Participatory Forest Management (PFM) and supporting NTFP trade. PFM agreements were crafted following boundary setting and negotiating roles and responsibilities. These agreements, with government as a signatory, formalized local rights over the forests, but within certain limits. For example, each community must commit to not converting forest to farmland.

The project helped honey producers to access new and larger markets, through the establishment of farmer PLCs^7^and honey co-operatives. These became focal points for Addis Ababa-based buyers who then provided training to farmers concerning harvesting methods, quality assurance, and storage. These value chain interventions resulted in Ethiopian honey being exported to Europe for the first time.

These initiatives achieved a growth in honey trade of 500% from 50 tonnes in 2005 to 300 tonnes in 2012 (Abebe 2013). In 2014, 500 tonnes of honey were traded by groups and co-operatives, with an unknown volume traded in other channels (Lowore 2014). Overall Abebe (2013, p 12) concluded that there had been a “big leap in supply of honey by producer groups and traders from the area to national and international markets through project facilitated market linkages.” The market price for honey rose from ETB 5 ($0.6 cents) to ETB 50 ($2.50) per kilo, an increase well exceeding the rate of inflation supporting the claim that, “The project has had a positive impact on the local honey trade. This NTFP trade is now well established and likelihood of long term benefits are high”, (NTFP-PFM 2013, p 35).

In terms of livelihoods, honey is one of the highest earning NTFPs with 97 households out of 115 reporting that at least 34% of their household income is derived from forest honey (Bekele and Tesfaye 2013).

Regarding forest conservation, community members reported a notable fall in forest encroachment and illegal harvesting and a notable increase in forest regeneration and healthy young seedlings. Before the project, 8.7% of respondents said forest regeneration was moderate or high, but afterwards 100% said forest regeneration was moderate or high (Bekele and Tesfaye, 2013). These changes led the project evaluator to report, “This is a substantial achievement and has potential to reduce the risk of deforestation in the area” (NTFP-PFM 2013, p 7).

The evidence from the project facilitates the examination of forest honey against the factors presented in Table 2.

## Forest Honey Analysis: Success/Failure Factors

### Inferior

Inferiority concerns product perishability, seasonality, and economic inferiority. Neither honey nor beeswax are highly perishable and can be bulked at collection centers with no time constraints or specialist storage required. It can be accumulated in economically viable volumes for transport. Project area buyers stipulate a minimum volume of 5 tonnes (one lorry load). Its non-perishability means that beekeepers not in immediate need of cash can store it until they need the money. The project found richer farmers tended to store honey, while the poorest sold it quickly, a pattern observed in other Ethiopian studies (van Biejnen et al. 2004; ILRI 2013). In this respect, honey compares well with other NTFPs, such as bush mango *Irvingia spp* (Nigeria) and *Gnetum* leaves (Cameroon and Nigeria), which are often wasted because of poor storage and inadequate transport, of which some transporters, knowing the urgency of sales, take advantage (Babalola 2009; Ingram et al. 2012).

The seasonal nature of NTFPs means that harvest times can be unpredictable, and income confined to limited periods. This presents challenges for poor families, although the non-perishability of honey alleviates this to some extent. However, while steady and predictable income may be preferable to seasonal income, it is better than none. Additionally, many high-value cash crops are seasonal, including coffee, cocoa, and pineapples, and this is not seen as a disadvantage.

Inferiority can also refer to products that are rejected as incomes rise. African forest honey is not an inferior good and is highly valued in most societies by rich and poor alike (Bradbear 2003; Ingram 2014). Honey harvested in SW Ethiopia, finds markets throughout Ethiopia, the Middle East, and Europe. The lack of pollution in SW Ethiopia means produce from this area is free from chemical contamination, and is consequently in demand by European high-value markets (Ingram and Njikeu 2011; David Wainwright 2016, personal communication).

### Substitutability

Some NTFPs can be readily substituted by alternatives. For example, vegetable ivory (*Phytelephas macrocarpa)* can be replaced by plastic (Barford et al. 1990). Sills et al. (2011) discuss how culture can maintain the value of NTFPs, and this might partly explain why forest honey is not readily substituted by sugar in Ethiopia. The Ethiopian tradition of making *tej* from honey also maintains high national demand. The authenticity of Ethiopian honey is highly appreciated in the Middle East and in Europe Ethiopian forest honey successfully competes with Chinese mass-produced honey. It performs well in specialist niche markets where it is sufficiently differentiated on taste, freedom from contamination, organic status, and authentic ‘‘natural’’ back-story.

### Unmanageable

NTFPs are natural products and since quantity, harvest time, and location are unpredictable and hard to manipulate, returns on labor can be low. However, this is not the case for forest honey. Forest beekeepers can increase the number of nest sites by placing more hives. Since hive ownership confers ownership of the honey harvest, beekeepers can rely on their harvest (except in the rare cases of theft). No time is wasted looking for wild nests, and so harvesting time can be managed (Bradbear 2009).

### Elite Capture

When a resource gains value, elites who previously had no interest in the product can take over extraction, processing, and trade (Dove 1994). In SW Ethiopia, access to the forest and specific trees does vary between ethnic groups and families (Hartmann 2004), but this is not a new phenomenon. There are few barriers to entry with youngsters embarking on beekeeping before they leave school (Bees for Development 2017). Hive ownership is a wealth indicator, but even the poorest have some hives, which are easier to accumulate than other wealth indicators (van Biejnen et al. 2004). For many households, honey is one of the most important sources of income: 70% of households derive some income from honey (Bees for Development 2017). While the benefits of honey trade are not equally spread, this does not present a clear case of ‘‘elite capture’’ following NTFP commercialization.

### Poverty Traps

Belcher and Schrekenberg (2007) classify NTFP activities as poverty traps where decreasing prices lead to increased harvesting to maintain income. There is no evidence that this applies to forest beekeeping. On the contrary, beekeepers invest more in beekeeping as the prices rise and the number of hives owned is positively related to wealth (van Biejnen et al. 2004). Income from honey increased during the project period (Bekele and Tesfaye 2013) and hive ownership increased by 70% between 2006 and 2016 (Bees for Development 2017).

### Over-Exploitation

NTFPs can be subject to over-exploitation. For example, the commercialization of Cameroonian *Prunus africana* bark led to degradation of the resource base and the ‘‘bread tree’’ (*Encephalartos cerinus*) was so depleted it is now subject to CITES trade prohibition (Stewart 2003; Donaldson 2008). Forest beekeeping does not cause resource degradation; the primary resource is nectar. Honey bees are merely agents, transforming nectar (a readily replenished plant product) into honey. Even where total cropping is practiced (when all the honey is taken, causing the bees to abscond) there is no evidence that the bee population is threatened. In fact, as honey demand increases, beekeepers place more hives in the forest, which is likely to increase the survival rate of swarms, although this has yet to be studied.

### Comparing Forest Honey Against the Success Factors

#### The natural resource base must be abundant

Cunningham (2011) argues that NTFPs can be successfully commercialized only when the natural resource is abundant, citing the successful commercialization of baobab (*Adansonia digitata)* and marula (*Sclerocarya birrea)*. The honey bee, *Apis mellifera* is found widely across the whole of Africa (Crane 1999) and feeds on many flowering plants (Fichtl and Adi 1994; Latham 2005; Bradbear 2009). Provided there are flowers and cavities (natural or human-made), bees will live in a wide range of habitats and the African population remains intact and healthy (Bees for Development 2013a; Bradbear 2009; Dietemann et al. 2009).

#### Sustaining a market requires quality, quantity, and timeliness

Commercial markets have demanding expectations regarding quality, quantity, and timeliness of supply and in their absence the potential of NTFPs will be limited (Ingram and Njikeu 2011). Beekeepers in the project area initially had difficulties meeting the market expectations of EU buyers. The project improved quality through interventions in the value chain by responding to concerns regarding the use of goatskins and fertilizer bags for storing honey, by providing plastic buckets. Growing demand has been met by beekeepers increasing their harvest and since forest honey supply is relatively elastic, they can continue to do this.

#### Upgrading within value chains

Adding value can be important for the success of NTFP trade (Meaton et al. 2015), but it is not always necessary for primary producers to do so. Opportunities to add value to forest honey include separating honey from wax, packaging, retailing, and even developing secondary products. In SW Ethiopia these opportunities are relatively limited. Most beekeepers sell the crude product with value addition occurring downstream. For example, the Ethiopian firm Beza Mar Ltd. separates honey from wax, it is ultra-filtrated in the UK, and The Body Shop uses the honey in their high-value products. This has increased demand with beekeepers benefitting without having to invest in time-consuming or capital-expensive interventions (Lowore 2014; Bees for Development 2017).

#### Clear rights to land

Tenure and access are key issues for commercializing NTFPs. In SW Ethiopia the ‘‘*kobo*’’ system, a long-standing inherited, customary tenure arrangement exists in two forms. In tree-*kobo*, individual trees are claimed for hanging hives, while land-*kobo* refers to an area of forest, which may contain many trees for hive-hanging. This privatization of a common resource appears to positively influence forest management. “In kobo ……. trees are properly managed and promising trees that could be a good nest tree will be tended and protected from damage. Beekeepers remove less vigorous trees to avoid competition on potential hive-hanging trees. Maximum protection is made to avoid damage to standing trees while felling trees for hive making or other purposes” (Endalamaw 2005, p 51). Increasing demand for honey in the project area has resulted in families re-asserting their claims over their *kobo* forest areas (Bees for Development 2017). This re-assertion of customary rights is related to Neumann and Hirsch’s (2000) observations that trade that develops in the ‘‘absence of existing controls of access’’, is least likely to be sustainable. The *kobo* has been overlain by the introduction of PFM and it is hard to disentangle these overlapping forms of tenure. PFM arrangements are recognized by government and give protection from private investors, so securing multiple forest benefits, including honey (NTFP-PFM 2013). The interaction between these external and local governance arrangements of the honey forests of SW Ethiopia are well described by Wiersum and Endalamaw (2013).

#### Local self-sufficiency

According to Cunningham (2011) if NTFPs are to be successfully commercialized, domestic self-sufficiency should be maintained. Brasileiro (2009) reported how commercialization of the Acai berry caused the price to rise beyond the reach of local people. There is no evidence that domestic honey use is undermined by commercialization in the project area, since domestic use is relatively low compared to the volumes traded (Melaku et al. 2014).

#### Conflict resolution mechanisms

The honey trade in the project area has experienced some conflicts and difficulties. For example, the Mejengir^8^honey co-operative did not succeed as a result of internal conflicts and misunderstandings with their honey buyers (Freeman 2012). However, overall trade is resilient, and even where project-designed structures fail, trade continues. “Experience from the NTFP-PFM project has shown that various actors will innovate once the market situation changes. It will change anyway” (Freeman 2012, p 11).

#### Price incentives must be right

The abundance of NTFPs in ‘‘good’’ years can depress prices and incentives to harvest. This can be prevented by growing product demand outside the local area and having enough buyers with working capital to buy the unusually large volumes on offer. Although honey supply has increased, the demand is coming from international buyers and has not resulted in lower prices; rather the reverse. Honey prices in the project area rose from 5 ETB ($0.6 cents) per kg in 2005 to 50 ETB($2.50) in 2015, a rise well beyond the rate of inflation largely due to linkages to Addis Ababa-based buyers and their contracts with overseas markets.

#### Building on existing markets, local markets

Commercialization of NTFPs is more successful when built on existing markets. The honey trade in SW Ethiopia has been established for many decades (Westphal 1975). The project strengthened an existing knowledge base and helped honey producers respond to the new buyers’ quality requirements by modifying harvesting and storage methods. The presence of pre-existing markets also reduces risk. When one project-supported marketing group had difficulties with their export buyers, they were able to sell the honey to local traders, and consequently did not suffer catastrophic loss (Lowore 2014). Honey that fails to meet export quality standards can be sold into other end markets. Sills et al. (2011) highlights the importance of local markets, an attribute enjoyed by forest honey.

#### Visionary champions make a difference

Visionary champions often play an important role in the development of new products and markets. For example, the success of the *rooibos* tea industry can be traced to Dr. Pieter Le Fras Nortier (Joubert and de Beer 2011). The African honey trade has similarly benefitted from industry champions. The first European buyer of honey from the project was Tropical Forest Products Ltd (TFP) founded by David Wainwright, who was determined to market African honey in the UK (Wainwright, 2002). He worked hard to convince UK customers to buy African honey, overcoming doubts about the marketability of its stronger taste (Traidcraft 2007).

#### Niche markets can reduce competition

Globally honey prices are highly competitive (Bees for Development 2006) and to compete, African honey needs to stress its ethical, natural, and environmental credentials. These features differentiate it from cheaper mass-produced Chinese honey. The use of project honey by The Body Shop evidences the success of this approach. A winning product needs to be quite special (to reduce competition), but not too special that it cannot be produced in sufficient quantities or so novel as to be un-marketable at scale. African forest honey meets this ‘‘goldilocks’’ ‘‘quite special but not too special’’, characteristic.

#### The power of strategic partnerships

Natural products sold into distant markets have complex trade requirements that are hard for farmers to negotiate (Ingram and Njikeu 2011). Strategic partnerships can overcome this. In 2005, 3 years before the first export of Ethiopian honey to Europe, Tropical Forest Products Ltd^9^and Beza Mar^10^, attended an African honey trade workshop organized by Bees for Development (Bees for Development 2005). In 2008, Ethiopia achieved EU ‘‘third country’’ listing with support from SNV^11^(Desalegne 2011), which meant that Ethiopian honey was eligible to be imported to the EU. Without these partnerships, access to this market would have been impossible. More locally the project forged links between producers and marketing organizations, established trade links, and strengthened the bargaining capacity of producers.

#### NTFP trade must make the forest more valuable than the alternative land use

Even if a NTFP has a market with positive impacts on livelihoods, the alternative land-use options must be understood. An economic analysis of land-use options in the project area showed that forests modified for coffee production yielded $547 per hectare, agriculture generated $303 per ha and sustainable forest management, including honey, generated $68 per ha, leading to the conclusion that “The limited revenues achieved from most NTFPs … leaves the … forest uncompetitive and encourage communities to engage in forest clearance. Hence … doubt can be cast on the ‘‘conservation by commercialization’’ hypothesis…” (Sutcliffe et al. 2012, p 479). Ingram’s (2014, p 205) research in Cameroon concurs, “the opportunity costs of other forest uses (for agriculture, hunting, grazing, fuelwood, and Prunus africana bark harvesting) are too high for apiculture chain actors to compete with.”

However, these analyses focus on economic returns from land, not the activity. Where capital and/or labor is in short supply and forest land is abundant then local people will also strongly consider returns on investment of cash and time (Kusters 2009). Endalamaw (2005) reported that forest beekeeping was not considered labor or capital intensive compared to other land-use activities and that beekeepers recognize its economic advantages. Hanging hives requires no capital, and can even be undertaken by teenage boys with no land or other assets of their own (Bees for Development 2017). Wainwright (1989) similarly found that forest beekeeping yielded good returns on time invested compared to other activities.

### Additional Factors

#### Biological characteristics of the NTFP determines likelihood of sustainable harvest

Neumann and Hirsch (2000) argue that there is greater potential for sustainability where NTFPs are fast-growing, fast-reproducing, and where harvesting does not impinge on reproductive potential. Honey production performs in this regard. Nectar harvesting has no negative impact on the plants and pollen transfer resulting from foraging is an essential ecological service. African honey bees reproduce easily so even if a colony is lost during honey harvest, it is likely to have already produced several swarms, easily compensating for losses.

#### NTFP specialization, forest modification, and biodiversity

There is increasing evidence that modifying the forest to favor NTFPs is the norm (Sills et al., 2011) and can yield enhanced incomes, but may impact negatively on biodiversity (Ruiz Pérez et al. 2004; Kusters et al. 2006). Beekeepers engage in active management and modification of the forest, with 95% of a sample in the project reporting individual actions to increase bee forage and to favor bee trees, by removing lianas from seedlings, protecting trees from fire, and avoiding crushing seedlings when felling larger trees (Bees for Development, 2017). However, this forest modification enhances biodiversity since beekeepers know that a variety of tree species is required so that nectar and pollen are available at different times of the year; that hive-making materials are sustained; and that good hive-hanging trees are protected. Forest honey is therefore derived from multiple forest species and this further motivates beekeepers to maintain forest biodiversity.

#### Direct link between action and benefit

Elliot and Sumba (2012) discuss conservation logic, the link between livelihood gains and conservation action. The silviculture practices discussed above exemplify this. Beekeepers clearly perceive a honey-derived income benefit from these actions. Another linkage is created by financial support for PFM. A key step in the project’s PFM process was establishing Forest Management Associations (FMAs) responsible for managing demarcated PFM forest and upholding the PFM agreement. The costs incurred (paying for patrols and taking offenders to court) are often funded through contributions from the honey co-operatives, creating a direct link between honey income and forest conservation.

## Discussion

The evidence suggests that forest honey in SW Ethiopia does not suffer from the barriers that have caused other NTFP commercialization endeavors to stumble. Forest honey is a non-perishable, highly marketable, high-value product with demand in local, regional, and international markets. It has local uses, but when carefully harvested and handled, it commands high prices by international buyers who use it in value-added products. The pre-existence of trade, local controls, knowledge, and experience prior to the development of new market opportunities provide a spring-board for commercialization and the ability to respond to new market quality and quantity expectations. The nature of forest beekeeping itself affords protection from over-exploitation. Provided the forests remain, bees and nectar will be abundant, and beekeepers can respond to higher demand without eroding the resource base.

There is clear evidence that the project generated increased beekeeping income. Evidence from the project also identifies positive forest impacts, but it is not easy to attribute the slowing down, and in some cases the reversal of forest degradation, to honey trade alone. Bee-friendly silviculture, customary tenure (*kobo*) and PFM appear to support forest maintenance, yet the first two were being practiced long before the project, when deforestation was happening. The recent increase in honey trade coincides with the introduction of PFM, but it is too soon to make a causal link between this and strengthened silviculture and *kobo* practices. Sutcliffe et al.’s (2012) conclusion that NTFP income was insufficient to deter local communities from engaging in forest clearance, further underlines the importance of over-stating any such success.

Despite these cautions, it is clear that there is genuine community enthusiasm for PFM. Local communities can choose to adopt PFM or to continue with the *status quo*, where they have no locally devolved rights and the forest is essentially open access and vulnerable to the risk of investors being allocated forest for agri-business. Without PFM they risk losing access to forest resources, including honey. By agreeing to PFM local people gain tenure security and access to many forest products and environmental services. More work is required to understand how important honey income is in generating the widespread support for PFM and in order to do so, it must be considered ‘‘in relation to the overall context of land uses in the area’’ (Ros-Tonen and Wiersum 2005). An aspect of this is beekeepers’ willingness to support forest conservation through FMAs by financial contributions from honey co-operatives and individual households. This constitutes direct and tangible evidence, linking honey production to forest conservation. It supports the argument that beekeeping plays a role in the economic calculation local communities make when deciding to adopt PFM since honey-derived income offsets some of the opportunity costs of accepting the restrictions which PFM brings, and helps pay for some of the direct costs.

### Reviewing Success and Failure Factors

Some of the factors identified appear to be less important, not only to honey, but to other NTFPs. Seasonality, for example, is not an insurmountable barrier to NTFP commercialization. Self-sufficiency is similarly a largely misplaced concern. NTFPs important for local use, e.g., firewood, are unlikely to have high commercial value, and for products traded to distant markets, subject to high-quality standards, there will almost always be a portion of the crop that is rejected and can be used in local markets or for domestic use. Niche markets can be ‘‘double-edged swords’’ in that they may need to be created, with significant marketing, and can be vulnerable to ‘‘faddish’’ changing tastes (Belcher and Schrekenberg 2007), but honey is unlikely to be susceptible. Part of the success of African forest honey is attributable to its characteristics in that it is a special product, but well recognized, niche, but not too niche!

NTFP trade is most likely to lead to the dual outcomes hoped for when the conservation logic between action and benefit is strong, ‘‘doable,’’ and delivers gains within an acceptable timeframe for poor people. The evidence in this paper suggests that honey delivers on all these points. Beekeepers know that tree loss leads to nectar loss leading to loss of income. They know what to do to prevent this and can see the immediate benefits of their actions, with a minimal time gap between action and benefit. A possibly unique characteristic of forest beekeeping is the ability to own a wild resource within a natural landscape. The transaction costs of managing common resources can be high, and the unpredictability of wild harvest can undermine returns on labor investment. These problems (associated with honey hunting), are overcome by beehive ownership. The placing of beehives affords ownership over the bees that choose to settle there, and the honey they subsequently store. This ownership is universally understood. This simple and inexpensive action removes uncertainty, reduces time-costs, and overcomes the unpredictability of honey hunting, and is a key reason for the economically rewarding nature of forest beekeeping.

## Conclusion

SW Ethiopia forest honey delivers important income for forest communities, and forest beekeepers are motivated to undertake actions that help maintain forests. Benchmarked against the factors influencing the success or failure of NTFPs, African forest honey performs exceptionally well. Key to this success was the pre-existing honey trade, the high-value nature of the product, and its appreciation in a range of markets. These afford producers flexibility and broad opportunities. Quality control improvements and the development of international trade links have driven both demand and price. Furthermore, forest beekeeping is sustainable and does not undermine the reproductive capacity of the bees, or the plants on which they feed. However, although these factors suggest that forest honey trade has considerable potential to deliver both livelihood and conservation outcomes, it would be unwise to claim that honey alone can halt forest loss. PFM clearly played a role in the success of honey trade in the project area and honey income in turn contributed to the broad support of PFM. This mutually supportive relationship requires more detailed examination so that the synergies it generates can be more fully understood.

The success factors observed in the case of forest honey in SW Ethiopia are likely to apply to other parts of Africa where forest beekeeping is practiced. Forest beekeeping systems are well-crafted resource utilization systems that combine elements of management with wild harvesting. Ownership of simple beehives and the utilization of abundant natural resources combine to offer an efficient and profitable livelihood activity that also has the potential to deliver on sustainable forest management. However, forest honey is not necessarily a near-perfect NTFP. Evidence presented in this case study has shown that its contribution to livelihoods and forest conservation has to be undertaken with regard to past and present land-use practices. In this case, the historic precedence of demand and the more recent establishment of tenure through PFM are thought to be key. For forest honey to deliver on livelihoods and forest conservation in other parts of Ethiopia and Africa, a full understanding of the context of trade and land-use needs to be achieved.

## Footnotes

1 Set by farmers clearing land, or by hunters.

2 Non-Timber Forest Products-Participatory Forest Management Project.

3 The NTFP project concluded in 2013, but continued with new funding and a new name, REPAFMA.

4 Full title: Forest landscape sustainability and improved livelihoods through non-timber forest product development and payment for environmental services.

5 REPAFMA = REDD + Participatory Forest Management in south-west Ethiopia.

6 Tej is a honey-based alcoholic drink.

7 Private Limited Companies.

8 Local ethnic group, famous as skilled forest beekeepers.

9 Lead UK importer of African honey.

10 Beza Mar Agro Industry PLC, lead Ethiopian honey trade and exporter.

11 SNV Netherlands Development Organization.
